# Supplementary material for: Same-model and cross-model variability in knee cartilage thickness measurements using 3D MRI systems
Source: PLoS One. 2025 Jun 13;20(6):e0324912. doi: 10.1371/journal.pone.0324912 (PMC12165388; doi:10.1371/journal.pone.0324912)
Supplement: Attachment — Submitted filename: 3D-MRI Ethics Approval M2021-291 Plos 250102 Japanese.pdf [file pone.0324912.s003.pdf]

2022 年 4 月 4 日

研究責任者
　再生医療研究センター・教授
　　　関矢 一郎　　殿

東京医科歯科大学
医　学　部　長

審　査　結　果　通　知　書

標記のことについて、下記のとおり審査結果を通知します。

記

委員会	医学部倫理審査委員会
審査年月日	2022 年 3 月 22 日
申請種類	新規申請
受付番号	M2021-291
課題名	膝関節を対象とした三次元 MRI 画像の撮影間誤差の検証
研究期間	2022 年 4 月 4 日　～　2025 年 3 月 31 日
判定	承認
理由<br>又は<br>勧告	無し
備考	
